# Outcome of Open Distal Tibial Physeal Fractures: A Descriptive Report of Two Cases

**DOI:** 10.7759/cureus.104513

**Published:** 2026-03-01

**Authors:** Jeevan K Sharma, Akash Sharma, Abhinav Phogat, Anish Kumar, Sunil K (Choudhary)

**Affiliations:** 1 Orthopaedics, Asian Institute of Medical Sciences, Faridabad, IND

**Keywords:** epiphyseal plate, fractures, gustilo-anderson, open, pediatrics, salter-harris injury, tibia

## Abstract

Open distal tibial physeal injuries are rare but clinically significant due to their proximity to the ankle joint and the potential impact on future growth in skeletally immature patients. These injuries pose unique challenges, including a higher risk of delayed healing, premature physeal closure, infection, and long-term deformity. We report two pediatric cases of open Salter-Harris type II distal tibial physeal fractures associated with Gustilo-Anderson type III soft-tissue injuries. Both patients underwent urgent debridement, stabilization with K-wire fixation, and multidisciplinary soft-tissue management. At the two-year follow-up, fracture union and infection control were achieved in both cases; however, medial physeal closure occurred, resulting in mild coronal plane deformity. These cases emphasize the importance of prompt debridement, careful physeal handling, multidisciplinary soft-tissue coverage, and long-term surveillance for growth disturbances.

## Introduction

Physeal fractures of the distal tibia occur in the growing skeleton and are seen more frequently in adolescents [1]. The distal tibial physis is of particular clinical importance, contributing approximately 40% of tibial longitudinal growth and 15-20% of overall lower-limb growth; consequently, injury to this region carries a substantial risk of growth arrest, angular deformity, and limb-length discrepancy. Following the seminal work of Salter and Harris describing five types of physeal fractures [2], Rang [3] added a sixth type involving perichondral ring injury, and Ogden [4] later expanded the classification to nine types. In a Salter-Harris type II injury, the fracture traverses the physis and exits through the metaphysis while sparing the epiphysis; although often associated with favorable outcomes in closed injuries, the risk of growth disturbance varies depending on fracture pattern, displacement, and associated soft-tissue damage.

While isolated reports of open physeal injuries at other anatomical locations exist, reports specifically addressing open distal tibial physeal fractures with Gustilo-Anderson type III soft-tissue injury remain extremely limited. According to the Gustilo-Anderson classification, type III injuries represent high-energy open fractures with extensive soft-tissue damage, contamination, and potential periosteal stripping, with subtypes IIIA and IIIB differing in the adequacy of soft-tissue coverage [5]. The combination of physeal injury and severe soft-tissue compromise presents distinct biological and mechanical challenges, including heightened infection risk, vascular compromise, difficulty achieving stable fixation without further physeal insult, and increased likelihood of premature physeal closure. Furthermore, wound management and closure in type III injuries are particularly demanding due to the limited distal tibial soft-tissue envelope, contamination, and the potential need for staged debridement or flap coverage. In addition to fracture healing and infection control, restoration and preservation of ankle range of motion remain critical outcome measures in these injuries, as postoperative stiffness can significantly affect long-term functional recovery in the growing child.

This report describes two pediatric cases that are unique in presenting open Salter-Harris type II distal tibial physeal fractures associated with Gustilo-Anderson type IIIA/IIIB soft-tissue injuries, an uncommon and high-risk injury combination. These fractures are inherently prone to infection due to their open nature. We discuss the initial assessment, surgical management, soft-tissue handling strategies, and clinical outcomes of these complex injuries.

## Case presentation

Case 1

A 14-year-old male patient presented to the emergency department following a fall from a bicycle, sustaining trauma to his right ankle. Clinical examination revealed a 5×3×2 cm open wound over the distal aspect of the right leg with exposed bone and visible deformity. Distal neurovascular status was intact.

**Figure 1:**
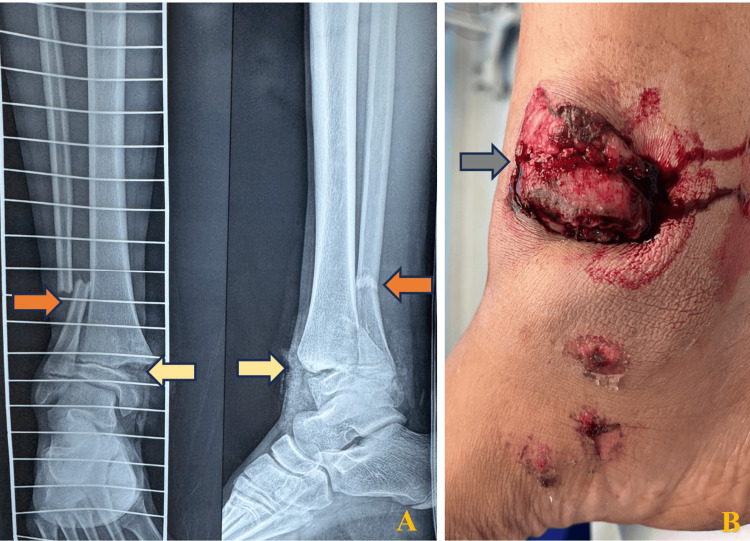
Anteroposterior and lateral radiographs (A) and clinical photograph (B) of the right ankle demonstrating a Salter-Harris type II distal tibial physeal fracture (yellow arrow) with a Gustilo-Anderson type IIIB open injury (gray arrow) and an associated distal-third fibular fracture (orange arrow). Arrows highlight the fracture line, open wound location, and fibular involvement

Plain radiographs demonstrated a distal tibial physeal injury consistent with a Salter-Harris type II fracture, associated with a distal fibular fracture (Figure 1).

The limb was splinted after the application of an impervious dressing, and intravenous antibiotics were given at the same time. Physeal injury location was thoroughly lavaged with six liters of normal saline. We were careful not to do curettage at the physeal site. Stabilization of the distal tibia was achieved using K-wire fixation, while the fibula was stabilized with a one-third tubular plate and screw (3.5 mm) construct.

**Figure 2:**
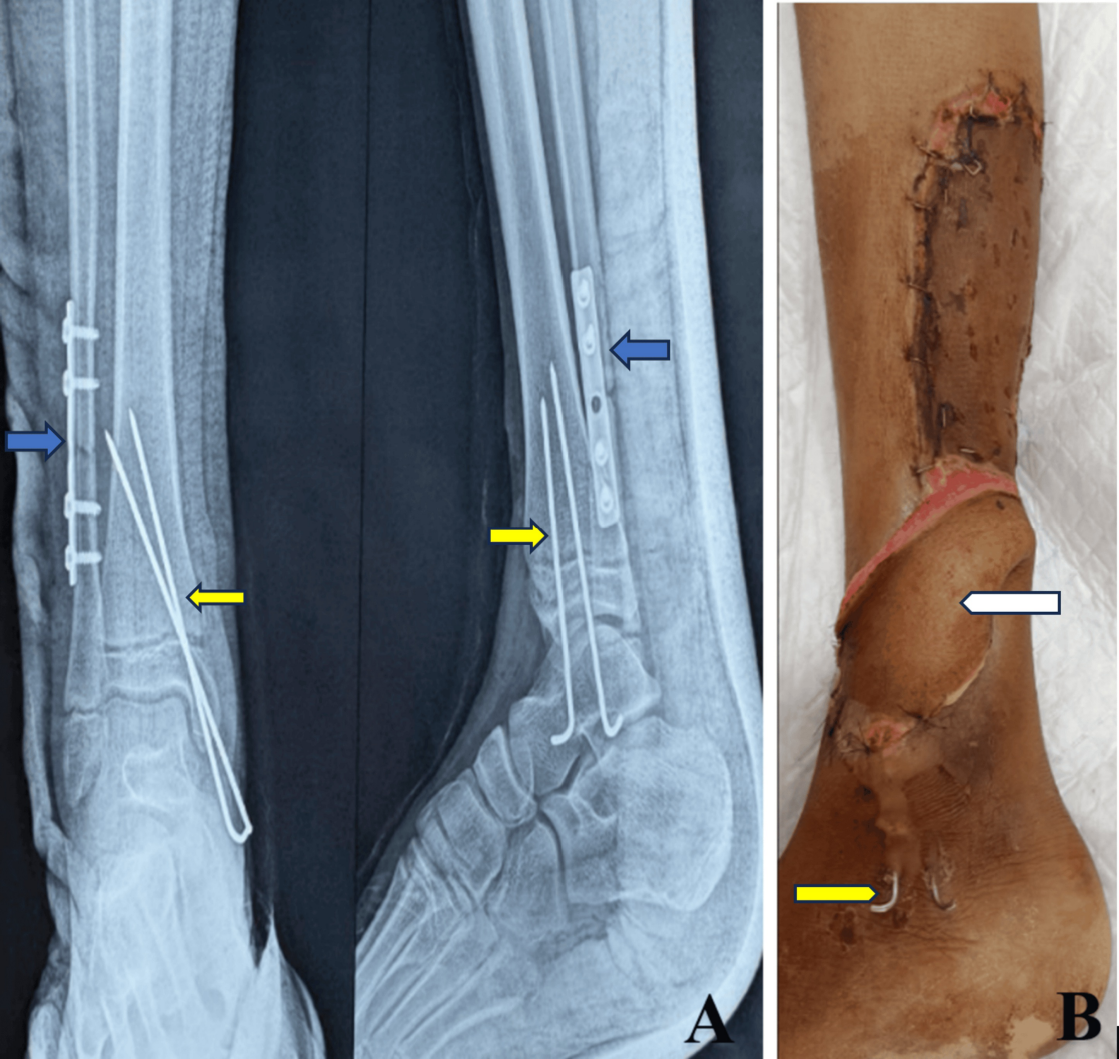
Immediate postoperative anteroposterior and lateral radiographs (A) demonstrating the reduction and internal fixation of the distal tibial physis with K-wires (yellow arrows) and the stabilization of the distal fibular fracture with a one-third tubular plate and screws (blue arrows). Clinical photograph (B) shows the postoperative lower limb with local fasciocutaneous flap (white arrow), split-thickness skin graft, and K-wires in situ. Arrows highlight key fixation and soft-tissue features

Injury was classified as grade IIIB on the Gustilo-Anderson classification. Plastic surgeons were involved to give a soft-tissue cover using a local fasciocutaneous flap, which was performed on the second postoperative day of the definitive fixation of bones (Figure 2).

The patient was followed at one month, six months, one year, and two years postoperatively. The local wound healed without infection or flap-related complications. Radiographs at the two-year follow-up demonstrated union of both the tibia and fibula.

**Figure 3:**
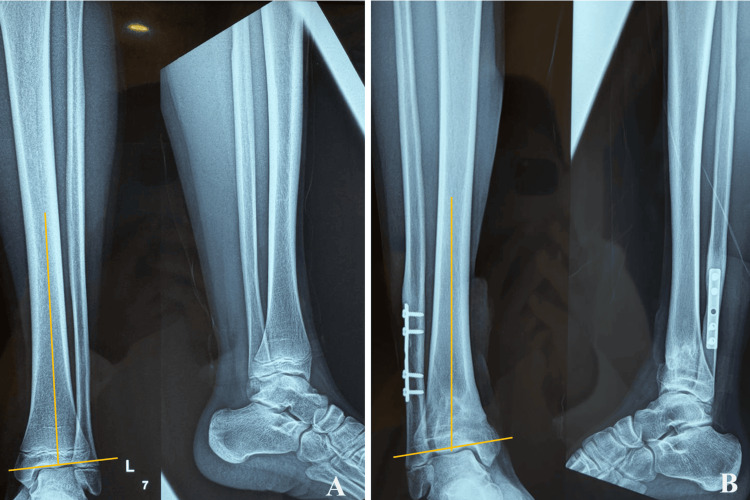
Anteroposterior and lateral radiographs at the two-year follow-up of the contralateral normal limb (A) and the operated limb (B) demonstrating the fracture union of the distal tibial physis with subtle varus deformity (4°), measured using the metaphyseal-diaphyseal angle (yellow arrow). No signs of infection are noted

**Figure 4:**
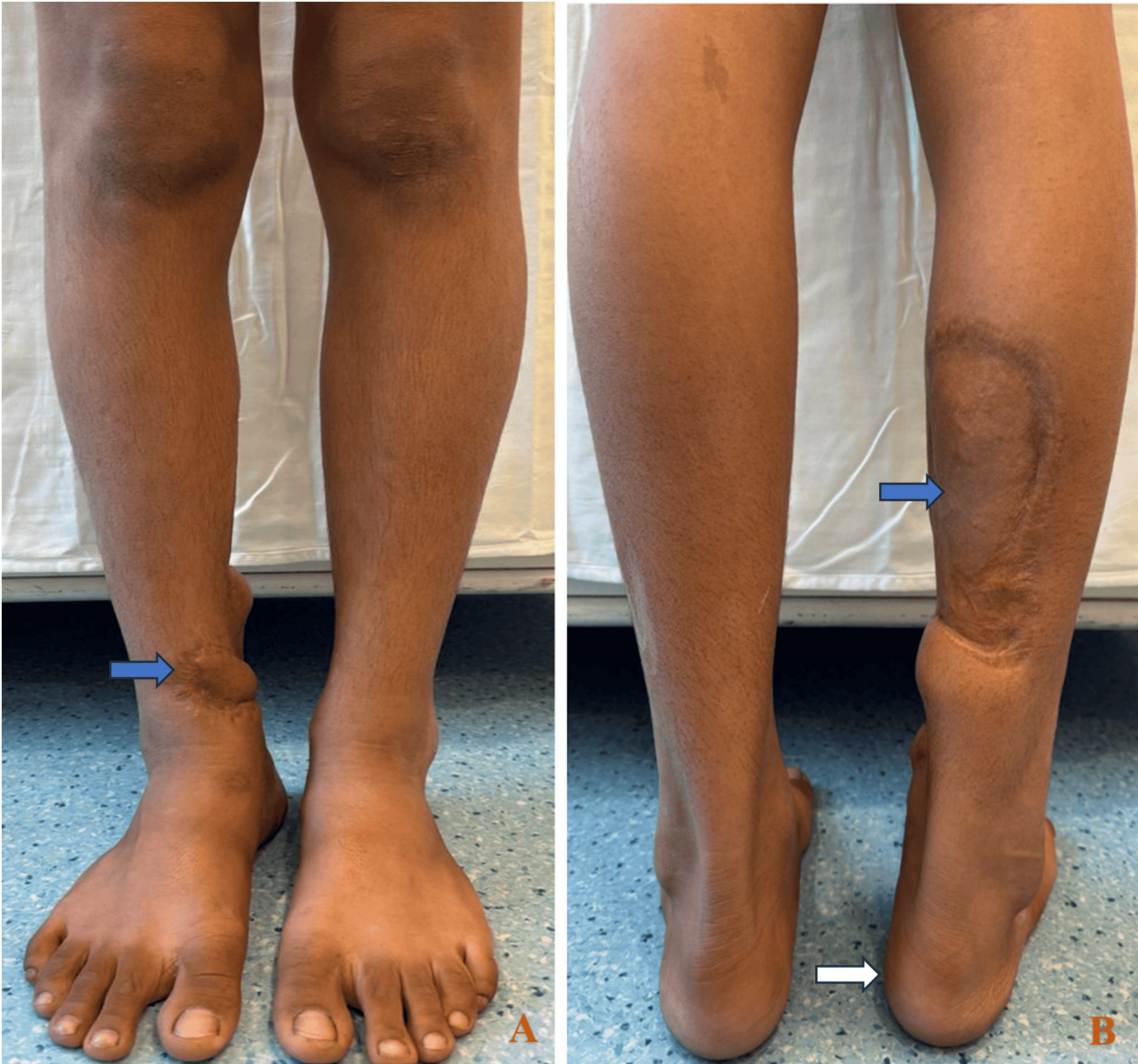
Clinical photographs of the operated limb from the front (A) and back (B) demonstrating a subtle angular deformity (white arrow) and a well-healed surgical scar with fasciocutaneous flap in situ (blue arrow). Arrows highlight residual deformity and soft-tissue healing

Physeal assessment revealed medial tibial physeal fusion. There was a tibia vara deformity in the coronal plane. The patient is asymptomatic, with full return to activities of daily living and the ability to participate in recreational sports (Figures 3-4).

Case 2

**Figure 5:**
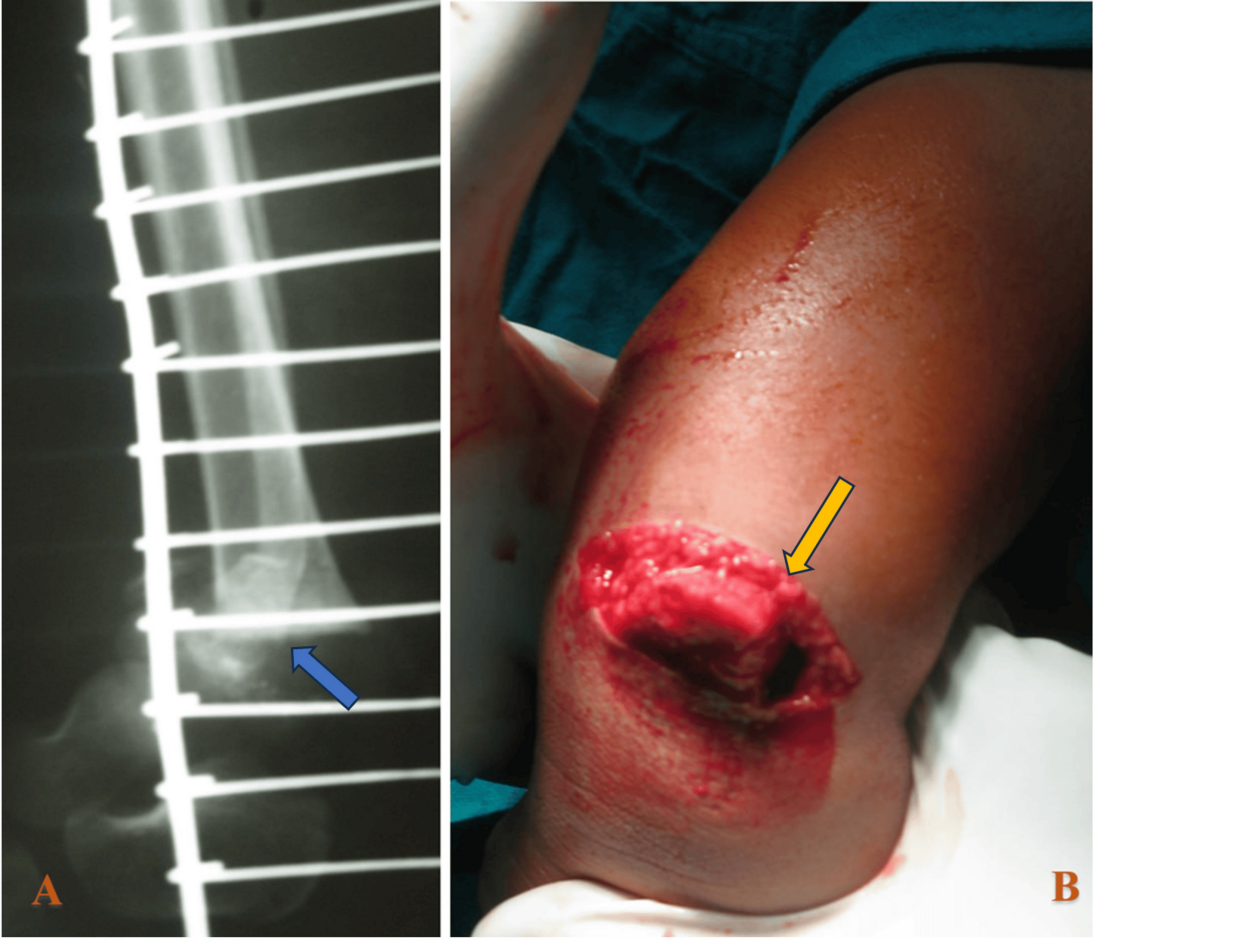
Lateral radiograph (A) and clinical photograph (B) of the left ankle demonstrating a Salter-Harris type II distal tibial physeal fracture (blue arrow) with an associated Gustilo-Anderson type IIIA open soft-tissue injury (yellow arrow). Arrows highlight the fracture line and open wound

A seven-year-old boy presented to the emergency department after a road traffic accident with a left ankle injury and wound. Initial assessment revealed a wound sized 4x2 cm with bone visible in it. Distal pulses were palpable, sensation over the foot was intact, and toe movements were fine (Figure 5).

Plain radiographs demonstrated a Salter-Harris type II fracture of the distal tibial physis, with posteromedial metaphyseal fragment displacement.

**Figure 6:**
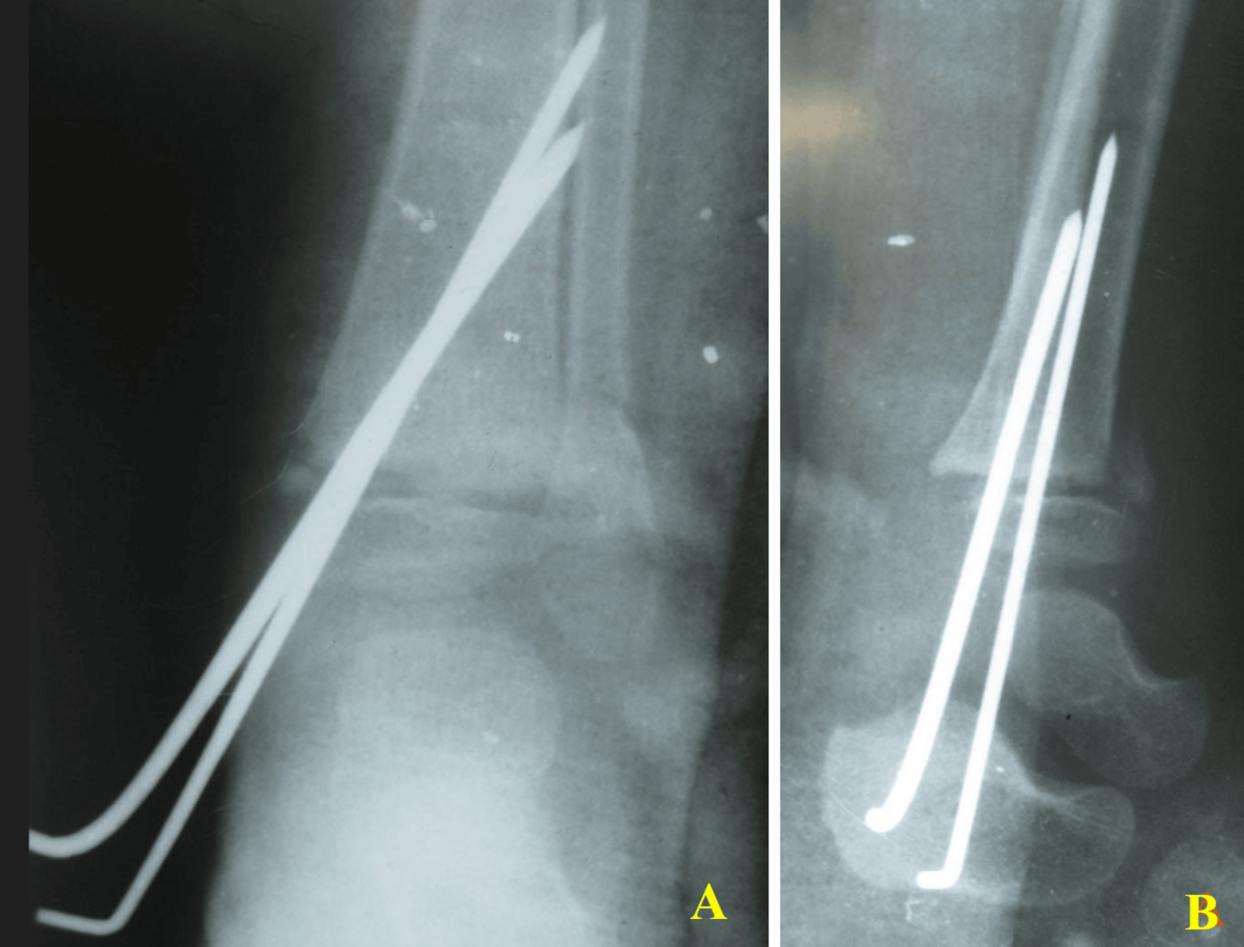
Immediate postoperative anteroposterior (A) and lateral (B) radiographs demonstrating fracture reduction and K-wire fixation

The patient was taken on an urgent basis to the operating theatre for thorough wound irrigation and debridement. After adequate cleansing and the removal of all devitalized tissues, the injury was classified as grade IIIA on the Gustilo-Anderson classification. The distal tibial physis was anatomically reduced under fluoroscopic guidance. Stabilization was achieved using K-wires, providing sufficient fixation while minimizing additional physeal insult (Figure 6).

Postoperative radiographs confirmed satisfactory alignment and stable fixation. The patient was maintained non-weight-bearing for six weeks, followed by progressive weight-bearing as tolerated.

**Figure 7:**
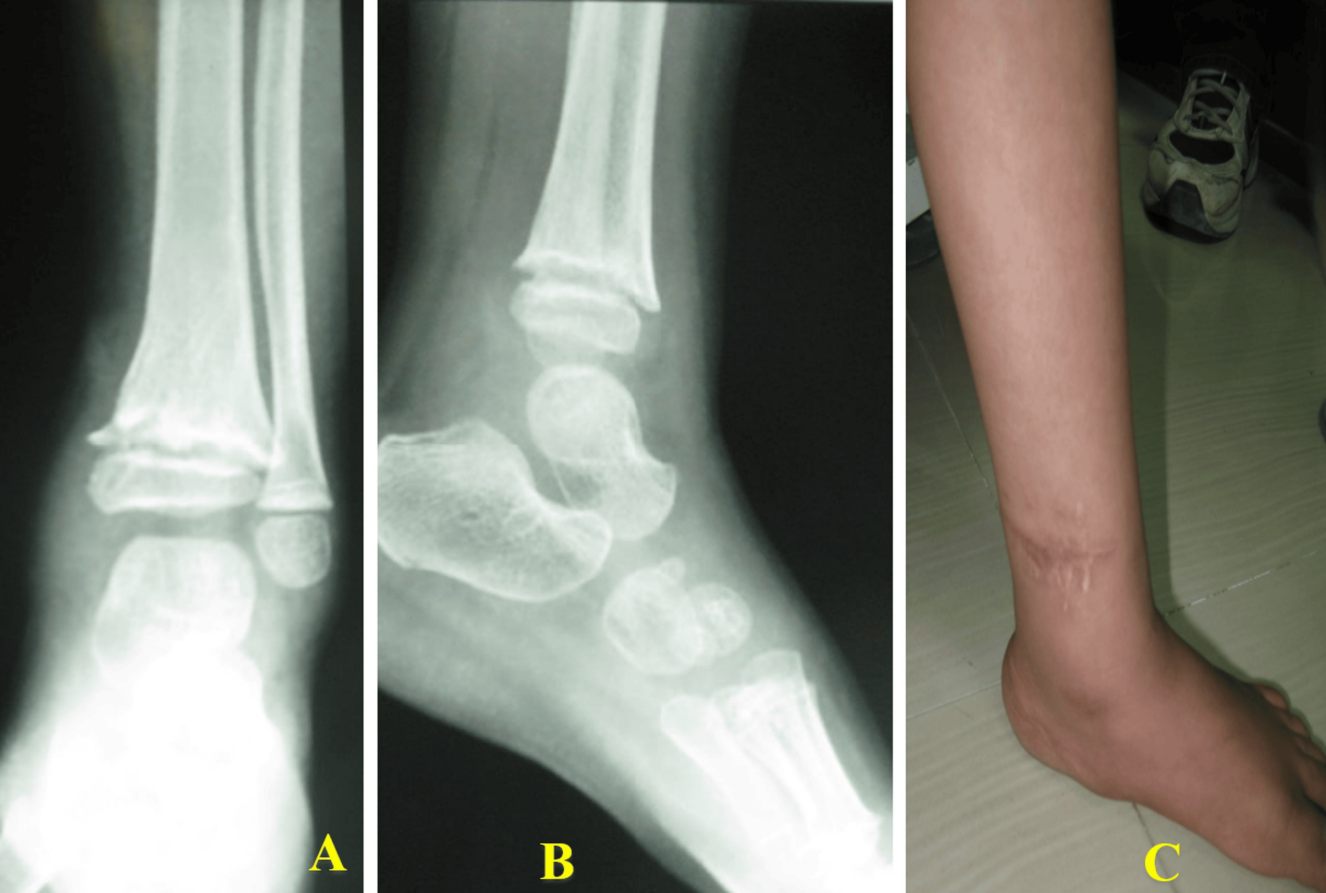
Anteroposterior (A) and lateral (B) radiographs, together with a clinical photograph of the healed wound (C), showing maintained alignment and no evidence of significant deformity or infection at two years postoperatively

**Figure 8:**
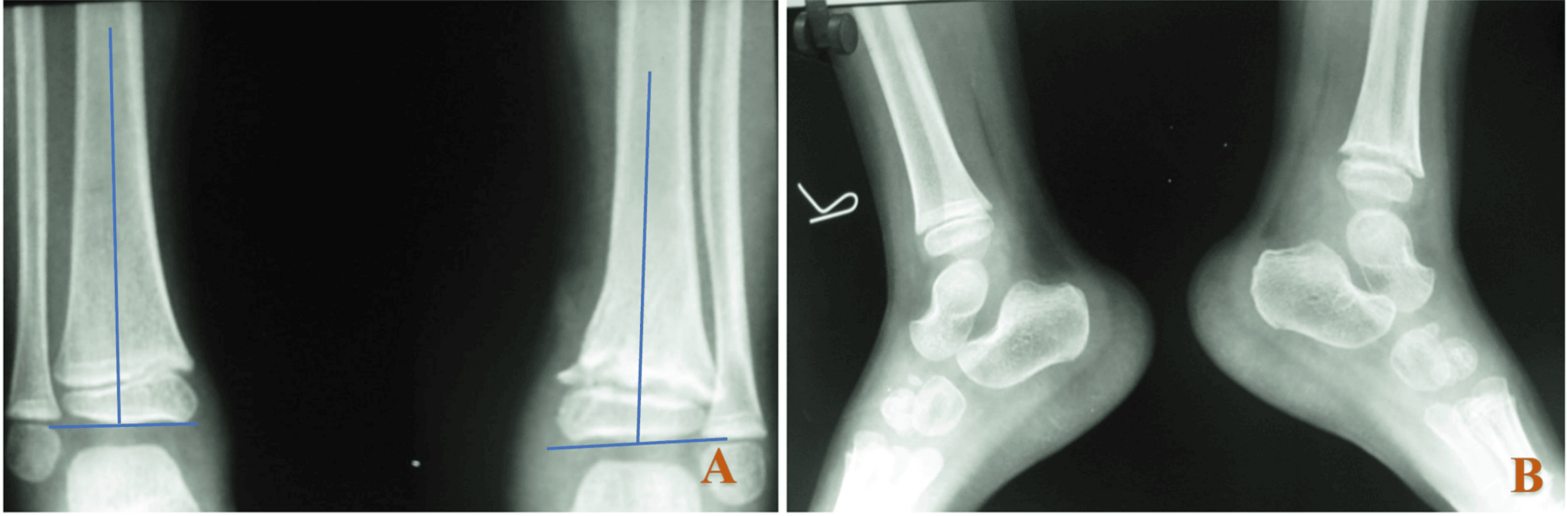
Anteroposterior (A) and lateral (B) radiographs at the two-year follow-up of the contralateral normal limb (right side) and the operated limb (left side) demonstrating fracture union of the distal tibial physis with a subtle varus deformity (2°) measured using the metaphyseal-diaphyseal angle (blue arrow)

At the two-year follow-up, the child remained asymptomatic with no pain on activity. Radiographs demonstrated early medial physeal closure, with coronal plane deformity. Clinical examination revealed a well-healed wound, full ankle range of motion, and normal gait (Figures 7-8).

The time interval from injury to surgical intervention was three hours in Case 1 and two hours in Case 2. Our antibiotic regimen consisted of cefazolin at a dose of 100 mg/kg/day administered in three divided doses every eight hours, along with gentamicin at 5-7.5 mg/kg/day, both continued for 48 hours. Definitive fixation was performed during the index procedure in both cases using parallel K-wire fixation of 2.5 mm in both cases. Preservation of the physis with K-wire placement was not feasible due to the inherent anatomical characteristics of the physis and the nature of the injury. The K-wires were retained for a duration of four weeks.

## Discussion

Childhood physeal fractures carry a risk of premature growth plate closure, which may lead to difficult-to-manage growth disturbances and related complications. Literature on lower-extremity physeal injuries and the factors predicting these outcomes remains limited. Historically, the chance of growth disturbances in physeal injury increases as the grade of injury increases on the Salter-Harris classification [6].

In evaluating pediatric patients with suspected open distal tibial physeal injuries, particular attention should be given to the presence of an open wound over the distal tibia, visible bone or deep contamination, deformity, swelling, and pain with restricted ankle motion. A meticulous neurovascular examination is essential, including documentation of distal pulses, capillary refill, and sensory function. Clinicians must also actively exclude associated complications such as compartment syndrome, vascular compromise, tendon injury, and ipsilateral fractures. Early recognition of these defining clinical features, combined with prompt radiographic assessment, is critical for the timely diagnosis and management of this uncommon but high-risk injury pattern.

Open physeal injuries are different than closed physeal injuries as they have chances of developing osteomyelitis and soft-tissue-related complications [7]. These injuries are likely to be high-energy injuries. Reported complications of distal tibial physeal injury include medial growth arrest, distal tibial varus, relative distal fibular overlength, and mild leg shortening. Management options for this complication are contralateral distal tibial and fibular epiphysiodesis to prevent significant limb-length discrepancy, completion of ipsilateral physeal closure, supramalleolar osteotomy with the insertion of a triangular cortical allograft to restore ankle alignment, stabilized with a medial locking plate, and fibular fixation using an intramedullary wire. Tibia vara is the most common complication requiring further surgery around the ankle in deformity of more than 10° [8-11].

There have been reports of open physeal injury of the great toe and their management and outcome [12], but reports on open distal tibial physeal injuries could only be found in one report with gunshot injury [13]. 

A literature review of previous studies on open physeal injuries, though heterogeneous, has been presented in Table 1. Although the present study focuses on distal tibial physeal fractures, the literature summarized in Table 1 demonstrates that open physeal injuries more commonly involve distal phalanges of the hallux and fingers, with only limited reports involving major weight-bearing physes. Among the included studies (1994-2024), most cases were Salter-Harris type I and II injuries occurring in pre-adolescent males, and the outcomes were generally favorable with appropriate treatment, showing minimal complications such as infection, physeal arrest, or deformity. In contrast, the single reported distal tibial physeal injury (Salter-Harris type IV) was associated with a more serious complication, highlighting the relatively higher risk profile of distal tibial involvement compared to distal phalangeal injuries. Similarly, distal femoral physeal fractures, another weight-bearing site, required longer follow-up due to concerns regarding growth disturbance. These findings corroborate the importance of early recognition and meticulous management of distal tibial physeal fractures, as injuries involving major growth plates of the lower limb may carry a greater risk of long-term sequelae compared to smaller, non-weight-bearing physes.

**Table 1:** Previous literatures on open physeal fractures

No.	Year of publication	Location of injury	Number of patients	Mean age (years)	Sex	Salter-Harris classification	Gustilo-Anderson grade	Outcome
1	2024 [13]	Distal tibial physis and talus	1	9	F	IV	Not mentioned	Non-union of the medial aspect of the distal tibia
2	2024 [14]	Distal femur physeal fracture	1	16	M	I	Not mentioned	Not mentioned
3	2021 [15]	Distal phalanx of the hallux	1	12	Not specified	I	Not mentioned	No physeal arrest, pain with weight-bearing, nor compromised normal function
4	2021 [16]	Distal phalanx of the hallux	5	10.3	M	II	Not mentioned	No patient complications (including infection) or reoperations at the two-month follow-up
5	2021 [17]	Ring finger distal phalanx	1	13	M	I	Not mentioned	Full recovery of the nail bed with a well-formed nail and no deformity of the phalanx
6	2019 [18]	Distal femoral physeal fracture	1	9	F	I	1	Followed clinically for two years and no angular deformity or leg-length discrepancy
7	2017 [12]	Distal phalanx of the hallux	1	12	M	II	Not mentioned	The patient went on to heal the fracture without any signs of infection
8	1994 [19]	Distal phalanx of the great toe	3	10.67	M	II	Not mentioned	No symptom or cosmetic defect

This report describes two cases that highlight several strengths. First, it addresses a rare and high-risk injury pattern of open distal tibial physeal fractures with Gustilo-Anderson type IIIA/IIIB soft-tissue injury in a pediatric population, for which existing literature is limited. Second, the report provides detailed documentation of multidisciplinary management, emphasizing the coordinated role of orthopedic and plastic surgery teams in achieving stable fixation and timely soft-tissue coverage. The case presentations in our report are structured to facilitate clinical recognition and reproducibility, outlining the mechanism of injury, detailed physical findings, radiographic characteristics, fracture classification, operative management, and longitudinal follow-up. Diagnostic warning signs include open wounds overlying the distal tibia, high-energy mechanisms, exposed bone, and radiographic evidence of physeal disruption with metaphyseal extension. Emphasis is placed on correlating early clinical findings with potential complications such as infection and premature physeal closure, thereby assisting clinicians in anticipating and monitoring for adverse outcomes. Fracture displacement, angular deformity, and soft-tissue injury severity were carefully documented to improve reproducibility and allow comparison with similar cases. Time from injury to operative debridement was recorded, and longitudinal follow-up included assessment for infection, fracture union, and signs of physeal arrest. 

Despite these strengths, several limitations must be acknowledged. The primary limitation is the small sample size, inherent to two cases, which limits generalizability. Second, the absence of a control or comparison group prevents definitive conclusions regarding the superiority of the chosen fixation or soft-tissue management strategies over alternative approaches. Third, although radiographic and clinical outcomes were favorable, advanced imaging modalities such as magnetic resonance imaging (MRI) were not used to assess subtle physeal bar formation, which may underestimate early or partial growth arrest. Additionally, functional outcomes were assessed clinically rather than using validated pediatric outcome scores, which could have provided more objective measures of recovery. The two-year follow-up may be too early to comment on future limb-length inequality or axial deformities. Lastly, only clinical assessment of the limb-length discrepancies was performed, and no radiological backup from scanograms was used.

## Conclusions

In these two pediatric cases of open distal tibial Salter-Harris type II physeal fractures associated with Gustilo-Anderson type IIIA/IIIB soft-tissue injuries, fracture union was achieved without clinical evidence of infection or osteomyelitis following urgent antibiotic administration, early operative debridement, stable fixation, and coordinated multidisciplinary care. At the two-year follow-up, mild coronal plane angular deformity consistent with partial physeal closure was observed, highlighting the susceptibility of the distal tibial physis to growth disturbance even with appropriate management. While the limited sample size precludes broader epidemiologic inference, these cases demonstrate that satisfactory outcomes, including preserved ankle range of motion and absence of deep infection, can be achieved when timely and meticulous surgical principles are applied. These findings contribute descriptive clinical insight to a rarely reported and high-risk injury pattern.
